# Enhancing Dental Material Performance: Tung Oil-Infused Polyurea Microcapsule Coatings for Self-Healing and Antimicrobial Applications

**DOI:** 10.3390/polym16070918

**Published:** 2024-03-27

**Authors:** Jiaqiao Zhong, Yuxiang Hu, Danqi Wang, Xingxin Zhou, Peiyu Yuan, Bowen Luo, Yuanzhe Li

**Affiliations:** 1 School of Medicine and Life Science, Chengdu University of Traditional Chinese Medicine, Chengdu 611137, China; jqiaozhong@163.com; 2 College of Design and Engineering, National University of Singapore, Singapore 119077, Singapore; e1143012@u.nus.edu; 3 Interdisciplinary Program of Biological Functional Molecules, College of Integration Science, Yanbian University, Yanji 133002, China; wang913621130@163.com; 4 Key Laboratory of Natural Medicines of the Changbai Mountain, Ministry of Education, College of Pharmacy, Yanbian University, Yanji 133002, China; 5 Zhuhai College of Science and Technology, Zhuhai 519041, China; jennyzhou@stu.zcst.edu.cn; 6 Melbourne Dental School, The University of Melbourne, Carlton, VIC 3053, Australia; peiyuyuan1209@gmail.com; 7 School of FESTU Transport, Dalian Jiaotong University, Dalian 116028, China; rocky_y200109@163.com; 8 School of Civil and Environmental Engineering, University of Auckland, Auckland 1010, New Zealand; 9 School of Materials Science and Engineering, Nanyang Technological University, Singapore 639798, Singapore

**Keywords:** tung oil-modified polyurea, self-healing coatings, dental material innovation, biocompatibility, mechanical durability

## Abstract

Within the realm of dental material innovation, this study pioneers the incorporation of tung oil into polyurea coatings, setting a new precedent for enhancing self-healing functionality and durability. Originating from an ancient practice, tung oil is distinguished by its outstanding water resistance and microbial barrier efficacy. By synergizing it with polyurea, we developed coatings that unite mechanical strength with biological compatibility. The study notably quantifies self-healing efficiency, highlighting the coatings’ exceptional capacity to mend physical damages and thwart microbial incursions. Findings confirm that tung oil markedly enhances the self-repair capabilities of polyurea, leading to improved wear resistance and the inhibition of microbial growth, particularly against *Streptococcus mutans*, a principal dental caries pathogen. These advancements not only signify a leap forward in dental material science but also suggest a potential redefinition of dental restorative practices aimed at prolonging the lifespan of restorations and optimizing patient outcomes. Although this study lays a substantial foundation for the utilization of natural oils in the development of medical-grade materials, it also identifies the critical need for comprehensive cytotoxicity assays. Such evaluations are essential to thoroughly assess the biocompatibility and the safety profile of these innovative materials for clinical application. Future research will concentrate on this aspect, ensuring that the safety and efficacy of the materials align with clinical expectations for dental restorations.

## 1. Introduction

The oral cavity is a challenging environment for dental materials, characterized by continuous exposure to mechanical stress from mastication, aggressive chemical exposure from dietary acids, and complex biological interactions with oral microflora. These factors can contribute to the degradation of dental restorations, leading to diminished integrity and shortened lifespan. The necessity for materials that can repair themselves in situ—self-healing coatings—has become increasingly apparent. Self-healing materials offer the potential to automatically repair micro-damage, extending the service life of dental restorations and reducing the frequency of dental interventions, thereby improving patient outcomes and reducing healthcare costs [1,2].

Polyurea coatings, with their superior mechanical strength, chemical resistance, and unique self-healing properties, have been identified as promising candidates for such applications. Originating from advanced materials science, these coatings undergo rapid polymerization reactions that create dense, cross-linked networks. This structural complexity confers significant protection against mechanical wear and chemical degradation, properties that are indispensable in the challenging oral environment. However, the application of polyurea coatings in dental restorations is not without its complexities. The distinct conditions of the oral cavity, characterized by constant exposure to mechanical stresses from mastication and a perpetually moist environment, present unique challenges for material integrity and performance. The intricacies of the oral environment underscore the necessity for dental materials that not only exhibit mechanical resilience but also maintain compatibility with the biological milieu. Despite their promising attributes, polyurea coatings must be optimized to enhance their biocompatibility and effectiveness in self-healing, particularly under wet conditions. Traditional self-healing mechanisms in polyurea are primarily designed for less aqueous environments, and their efficiency can be significantly compromised in the saliva-rich oral cavity. This limitation highlights a critical area for research and development—modifying polyurea coatings to thrive within the unique constraints of dental applications, thereby ensuring the longevity and integrity of dental restorations [1,3].

Against this backdrop, the strategic selection of tung oil as a modifying agent for polyurea coatings represents an innovative leap forward. Tung oil, derived from the seeds of the tung tree (*Vernicia fordii*), boasts a rich legacy in the realm of protective finishes for wood and metal, revered for its remarkable polymerization capabilities. Upon exposure to air, tung oil undergoes an oxidative polymerization reaction, transforming into a tough, impermeable film. This process, facilitated by the unsaturated fatty acids (primarily alpha-eleostearic acid) in tung oil, results in a cross-linked polymeric network. These unique molecular characteristics endow tung oil with exceptional water resistance and durability, making it an ideal candidate for enhancing the protective qualities of polyurea coatings in dental applications [4,5]. The intrinsic properties of tung oil present a multifaceted solution to the challenges encountered by polyurea coatings within the dental environment. Its formidable water resistance is critical in thwarting the penetration of moisture—a prevalent and persistent threat to dental materials exposed to the saliva-rich oral cavity. Moreover, tung oil’s capacity to form a robust microbial barrier leverages its natural fungicidal and bactericidal properties, offering an additional layer of protection against microbial contamination, which is a common cause of dental restoration failure [6]. Beyond its protective attributes, tung oil’s synergy with polyurea’s self-healing mechanisms heralds a significant advancement in material science for dental applications. The integration of tung oil into the polyurea matrix can facilitate the autonomous repair of minor damages, such as scratches or cracks, that may occur during normal dental use. This self-healing process is powered by the dynamic interactions between the reactive sites within the polyurea network and the polymerizable components of tung oil. When damage occurs, the disrupted areas can reinitiate polymerization or cross-linking reactions, effectively ‘healing’ the material. This capability not only extends the service life of dental restorations but also maintains their integrity and functionality, preventing the ingress of bacteria and other deleterious substances.

The integration of tung oil into polyurea coatings represents a significant advancement in the development of dental materials. This innovative approach leverages the self-healing nature of polyurea and the protective qualities of tung oil to address the inherent challenges of the oral environment. By significantly enhancing both the mechanical robustness and biological compatibility of polyurea coatings, our objective is to markedly prolong the lifespan of dental restorations, minimize the frequency of replacement procedures, and elevate the standard of patient care. The significance of our study is twofold: Firstly, it provides a comprehensive analysis of how tung oil modification improves the performance of polyurea coatings under the specific conditions of the oral cavity. Secondly, it quantitatively demonstrates the enhanced durability, wear resistance, and microbial resistance of these modified coatings compared to traditional dental materials. Our findings underscore the potential of this innovative approach to not only extend the service life of dental restorations but also to offer a more sustainable and biocompatible solution, heralding a new era in the field of restorative dentistry. Ultimately, this research aims to transform dental material science, paving the way for the development of more durable, reliable, and patient-centric dental care solutions.

## 2. Developing Tung Oil-Infused Polyurea Coatings for Enhanced Dental Applications

### 2.1. Analysis of Self-Healing Mechanisms in Dental Polyurea Materials

Polyurea coatings, defined by their unique urea linkages—resulting from the reaction of isocyanates with amines—offer a compelling combination of mechanical properties that are desirable for dental applications [1]. The urea linkage, a strong hydrogen-bonding site, imparts high tensile strength and impact resistance [7]. The original document highlights the chemical robustness of polyurea, derived from its resistance to solvents and chemicals, which are prevalent in the oral environment. This resistance is crucial for materials continuously exposed to saliva, which contains a complex mix of chemical entities. In the realm of dental materials, polyurea coatings have garnered attention due to their impressive mechanical strength, elasticity, and chemical resistance, which are essential for protective applications. Moreover, polyurea’s rapid polymerization and formation of a robust network render it an excellent candidate for creating barriers against common dental material assailants, such as bacteria and acidic compounds [8]. Studies have indicated that the mechanical properties of polyurea coatings, including their ability to withstand significant strain and stress, make them suitable for withstanding the oral environment’s mechanical demands [3].

Two principal self-healing strategies have been identified: intrinsic and extrinsic [9,10]. Intrinsic self-healing operates on the molecular level within the polyurea matrix, exploiting reversible urea linkages and associated hydrogen bonding [11]. These bonds are dynamic, breaking and reforming, allowing the polyurea to mend autonomously when stressed or damaged. This characteristic is particularly beneficial for dental materials where resilience and longevity are paramount [12]. On the other hand, extrinsic self-healing mechanisms are characterized by the integration of microcapsules filled with a polyurea pre-polymer or monomer. Damage to the material triggers the rupture of these microcapsules, releasing their contents, which then react with the material or ambient moisture to seal cracks or breaks [13]. An illustrative example of an intrinsic healing event would be within a dental composite material where micro-cracks, which might develop over time due to regular mastication, would close up due to the self-realignment and reconnection of the polymer chains. For extrinsic healing, envision a dental crown that, upon experiencing a fracture, would benefit from the immediate release of healing agents from microcapsules, sealing the crack and potentially preventing the need for dental intervention [14].

Control methodologies for these self-healing mechanisms are engineered specifically for their respective systems. Intrinsic healing is modulated by the physical and chemical properties of the polymer, such as the balance between soft and hard segments, which influences flexibility and self-healing efficiency [15]. In contrast, extrinsic healing is mechanically activated by damage to the material, which necessitates a carefully designed microcapsule shell that remains intact under normal conditions but responds when needed [16]. Technological advancements in polyurea for dental applications are focused on optimizing these healing mechanisms. Intrinsic self-healing advancements aim to synthesize polymers that can effectively self-heal in the moist and chemically diverse oral environment. Extrinsic healing advancements concentrate on microencapsulation technologies that can withstand the unique challenges posed by oral applications, such as resistance to saliva and the mechanical forces of chewing [17].

### 2.2. Integration of Tung Oil into Polyurea Coatings

#### 2.2.1. Benefits of Tung Oil for Dental Coatings

Tung oil, sourced from the seeds of *Aleurites fordii*, is characterized by a high content of α-eleostearic acid, a conjugated linolenic acid [18]. This molecular structure imparts notable water resistance and the capacity to establish a defensive barrier against microbial infiltration—two essential properties for materials used in the moisture-rich and microbially diverse oral environment. A comparative analysis of the fatty acid composition of tung oil relative to other drying oils, such as linseed oil, reveals that tung oil’s high proportion of conjugated fatty acids leads to a densely cross-linked polymeric network upon oxidation and polymerization. This tightly cross-linked structure is responsible for its enhanced protective qualities [19]. Table 1 presents a detailed comparison of the fatty acid profiles of various drying oils, underscoring the unique chemical attributes of tung oil that contribute to its superior performance in dental material applications [20].

#### 2.2.2. Biocompatibility and Mechanical Properties in Dental Environment

The oral cavity is a complex environment where materials are exposed to various mechanical and biochemical challenges [21]. Tung oil’s biocompatibility is a significant advantage here. Derived from natural sources, it is inherently less reactive and more harmonious with the biological tissues found in the oral cavity. Unlike synthetic materials, which can degrade into potentially toxic compounds [22], tung oil is less likely to release VOCs or other harmful substances that could lead to inflammation, allergic reactions, or toxicity. This natural oil is metabolized by the body more readily, reducing the likelihood of negative long-term effects. Moreover, the oxidative polymerization of tung oil does not rely on harmful catalysts or byproducts that could leach out and compromise oral health. Instead, it forms a biologically inert network upon curing, which is less likely to provoke a biological response.

Dental materials must possess the strength to withstand the forces of mastication and the resilience to endure constant thermal fluctuations within the mouth. Tung oil-modified polyurea coatings exhibit an increased capacity to absorb and dissipate energy compared to their traditional counterparts. The presence of tung oil provides a balance of rigidity and flexibility [23], which is essential to resist cracking or chipping under the stress of biting and chewing. The enhanced elasticity and toughness of tung oil-infused polyurea also contribute to better wear resistance, an important characteristic for materials used in restorative dentistry (Table 2). These mechanical attributes are crucial for the longevity of dental restorations, as they must maintain their integrity over many years of service.

### 2.3. Advanced Microencapsulation Techniques for Tung Oil in Polyurea Dental Applications

#### 2.3.1. Characteristics and Functions of Tung Oil as a Healing Agent

Tung oil, sourced from the *Aleurites fordii* plant, is a natural drying oil that is prized for its polymerization capabilities. The oil’s fatty acid profile is rich in unsaturated bonds, especially α-eleostearic acid, which contains conjugated double bonds that are reactive toward atmospheric oxygen [18]. This reactivity is the cornerstone of the oil’s ability to form a dense, cross-linked polymeric network upon exposure to air—a transformation that underpins the self-healing attributes of tung oil-infused coatings [19].

#### 2.3.2. The Oxidative Polymerization Mechanism of Tung Oil

The oxidative polymerization of tung oil is a catalytic process facilitated by transition metals that enhance the rate of cross-link formation. The mechanistic details are as follows [20]:

- **Initiation**: Free radicals generated from the oil’s unsaturated fatty acids react with molecular oxygen, forming peroxyl radicals.

•R• + O_2_ → ROO•

- **Propagation:** These peroxyl radicals are highly reactive and can abstract hydrogen atoms from other unsaturated fatty acid molecules, leading to the formation of hydroperoxides and the propagation of further radical reactions.

•ROO• + RH → ROOH + R•

- **Decomposition:** The hydroperoxides can decompose, especially in the presence of transition metal catalysts, yielding alkoxy radicals and hydroxide ions, which then participate in further polymerization steps [21].

−ROOH + Transition Metal (e.g., Co^2+^) → RO• + Transition Metal (e.g., Co^3+^) + OH^−^

- **Termination (Cross-linking):** Peroxyl radicals add across carbon-carbon double bonds within other fatty acid molecules, leading to a cross-linked, stable polymeric network, which is crucial for the material’s self-healing properties.

ROO• + C=C → RO−O−C−C•

These reactions are integral to the development of the tung oil-enhanced polyurea’s self-healing capacity. They form a robust material that can repair itself, extending the lifetime and durability of dental restorations [20].

#### 2.3.3. Strategic Selection of Healing Agents and Microcapsule Materials

The deliberate choice of tung oil as the healing agent in polyurea dental coatings is predicated on its innate biocompatibility and its ability to enhance the mechanical robustness of the coatings. Tung oil’s unique chemical composition, rich in polyunsaturated fatty acids like α-eleostearic acid, allows it to undergo a rapid oxidative polymerization process [7,18,19]. This characteristic is especially beneficial in the oral cavity, where the material must remain inert and non-reactive against soft tissues yet capable of activating its self-healing mechanism when required. Furthermore, the polymeric network formed by polymerized tung oil has the dual advantage of being flexible enough to withstand the dynamic mechanical stresses in the oral environment and tough enough to provide a lasting protective barrier. This balance is crucial for dental applications, where the material must adapt to the continual pressure of mastication without compromising its structural integrity. In parallel, the selection of melamine–urea–formaldehyde (MUF) for the microcapsule shell is informed by its outstanding chemical stability, which is essential for ensuring the longevity of the microcapsules within the aggressive chemical milieu of the mouth [24]. The MUF shell must withstand daily exposure to a variety of chemical stimuli, including acidic and basic food substances, without degrading or releasing its contents prematurely.

The compatibility of MUF with both the polyurea matrix and the tung oil is another cornerstone of this selection. This compatibility ensures that the microcapsules can be seamlessly integrated into the polyurea matrix, providing an uninterrupted surface that is crucial for the aesthetics and functionality of dental coatings. The MUF microcapsules are engineered to respond to mechanical impact, which is an inevitable occurrence in dental applications. Upon such impact, the microcapsules are designed to rupture in a controlled manner, releasing the tung oil in a localized fashion. This targeted release is the trigger for the self-healing process, whereby the tung oil interacts with ambient oxygen and moisture to initiate the polymerization process. This process effectively seals micro-cracks or scratches, restoring the coating’s integrity and prolonging the life of the dental restoration.

#### 2.3.4. Integration of Tung Oil into Microcapsules for Polyurea Coatings

The encapsulation of tung oil within PUF microcapsules is a deliberate approach to preserve the healing agent’s reactivity. This encapsulation process is critical, as it ensures the longevity and efficacy of the self-healing mechanism. Upon damage to the polyurea matrix, such as scratches or microfractures, the microcapsules are compromised, leading to the release of tung oil. The oil then interacts with the moisture and oxygen in the oral environment to initiate polymerization, repairing the polyurea coating and restoring the protective barrier against microbial ingress and other potential dental material failures. Via this advanced microencapsulation technique, tung oil’s natural properties are harnessed to create a dynamic, responsive dental material. This represents a significant advancement in the development of self-healing dental coatings, with implications for the longevity and reliability of dental restorations.

## 3. Methodology

### 3.1. Materials

Melamine (M), urea (U), and formaldehyde (F) as the core reactants for MUF synthesis; melamine (99% purity, Sigma-Aldrich, St. Louis, MO, USA), urea (98%, Fisher Scientific, Hampton, NH, USA), and formaldehyde (37 wt% solution, Merck, Kenilworth, NJ, USA) for MUF microcapsules; Lauryl sulfate sodium (LSS) (99%, Acros Organics, Geel, Belgium) and Polyvinyl alcohol (PVA) (98–99%, Alfa Aesar, Haverhill, MA, USA) for stabilization; tung oil (100% refined, Tung Oil Producers, Wuhan, China) for the core material; Methylene diphenyl diisocyanate (98%, Dow Chemicals, Midland, MI, USA) and p-Phenylenediamine (>99%, BASF, Ludwigshafen, Germany) for polyurea synthesis; Tetrahydrofuran (>99.9%, VWR Chemicals, Radnor, PA, USA) and O-(2-Aminopropyl)-O′-(2-methoxyethyl) polypropylene glycol (95%, Huntsman Corporation, The Woodlands, TX, USA) as the reactive diluent. These materials, selected for their exceptional purity from established suppliers, are critical for the fabrication of microcapsules and the resulting polyurea resin.

#### 3.1.1. Synthesis of Melamine–Urea–Formaldehyde (MUF) Microcapsules

The MUF microcapsules were prepared using an in situ polymerization method within an oil-in-water emulsion, based on a technique adapted from the work of J. K. Lee et al. [11]. The synthesis followed an optimal molar ratio of melamine/urea/formaldehyde at 3:1:8.5. Initially, solutions of LSS and PVA were prepared; the LSS solution was heated to 70 °C for 20 min, and the PVA solution was similarly maintained at 70 °C, but for 2 h to ensure complete dissolution. An MF (melamine–formaldehyde) prepolymer was then produced by reacting melamine with formaldehyde in distilled water at room temperature (25 °C) for 25 min. Separately, urea was dissolved in distilled water and stirred vigorously. This urea solution was combined with the MF prepolymer, LSS, and PVA under increased agitation to ensure thorough mixing. Tung oil was then gradually added to the mixture. The entire emulsion was kept at 86 °C for 5 h with continuous stirring to facilitate the polymerization process. Upon completion of the reaction, the microcapsules were isolated via filtration and subsequently dried under ambient conditions to yield the final MUF microcapsules encapsulating the tung oil.

#### 3.1.2. Synthesis of Polyurea and Integration of Microcapsules

The polyurea matrix was synthesized from two primary reactants: Methylene Diphenyl Diisocyanate (MDI) for Part A and p-Phenylenediamine (p-PDA) for Part B. To prepare Part A, MDI was dissolved in Tetrahydrofuran (THF) to facilitate uniform mixing. Similarly, Part B was prepared by dissolving p-PDA in THF, with continuous stirring for one hour to ensure complete dissolution. These reactant solutions (Part A and Part B) were then combined in a 3:1 ratio, along with the reactive diluent, to initiate the polyurea formation process. Following this, the previously prepared melamine–urea–formaldehyde (MUF) microcapsules were uniformly dispersed into the polyurea resin. This dispersion was achieved using a mechanical mixer set at 200 revolutions per minute (rpm) for 5 min, ensuring an even distribution of microcapsules throughout the resin. The resulting composite material was then applied to a steel panel using a film applicator, ensuring an even coat. This coated panel was left to cure at room temperature for a period of 7 days to achieve optimal material properties. The detailed chemicals and their weight percentage are listed in Table 3.

### 3.2. Characterization

#### 3.2.1. Scanning Electron Microscopy (SEM)

Following ASTM E1508 [25], SEM analysis was carried out using a JEOL 6360LV (JEOL Ltd., Akishima, Japan) to observe the microstructure of the polyurea coatings with tung oil microcapsules (PUA-MCs). The SEM operated with a thermionic tungsten filament, providing a resolution of 4.0 nm and an accelerating voltage range from 0.5 to 30 kV. The magnification ranged from 50× to 100,000×, suitable for detailed surface analysis.

#### 3.2.2. Fourier-Transform Infrared (FTIR) Spectroscopy

In compliance with ASTM E1252 guidelines [26], FTIR spectroscopic analysis was employed to characterize the chemical constituents of the polyurea microcapsules (PUA-MCs). Utilizing a Frontier PerkinElmer spectrometer outfitted with a Universal ATR accessory, we performed a comprehensive analysis within the mid-infrared (mid-IR) spectral range. This robust analytical approach enabled the precise identification of functional groups integral to the chemical structure of both the tung oil and the polyurea matrix. The spectral scanning was conducted over a range of 4000 cm^−1^ to 400 cm^−1^ with a resolution of 4 cm^−1^. Each spectrum represents an accumulation of 32 scans to ensure optimal signal-to-noise ratio and data fidelity.

#### 3.2.3. Adhesion Test

To evaluate the mechanical integrity of the self-healing coatings, an adhesion test was conducted in accordance with ASTM D3359 standards [27]. This standard assesses the adhesion performance of coatings via a methodical application and removal of tape under controlled conditions. The tests were carried out using an MTS Criterion Model 42 Static Mechanical Tester (MTS Systems Corporation, Eden Prairie, MN, USA). The specimens prepared for testing were rectangular sections of the coated substrate, each measuring 75 mm in length and 25 mm in width. The substrate utilized was a standard steel panel commonly employed in coating adhesion studies to provide a consistent and uniform surface for adhesion. A load cell with a capacity of 50 N was employed to accommodate the expected range of forces associated with the tape removal process. The loading grip used for the test was a custom-fabricated clamp specifically designed to apply a steady, parallel force to the coated surface, mirroring the action of tape removal.

During the testing process, the crosshead speed was set to 5 mm/minute, a rate chosen to ensure consistent and gradual application of force, which closely simulates the stresses experienced during the manual removal of tape. This speed was maintained before and after the healing process to provide a comparative analysis of the coating’s adhesion. To ensure the accuracy of the adhesion measurement, the sample’s position was carefully aligned with the axis of the load cell to avoid any off-center loading. The uniform force applied by the mechanical tester allowed for a quantitative assessment of the coating’s adhesion, both pre-healing and post-healing, thus offering a reliable measure of the self-healing process’s efficacy.

### 3.3. Simulation of Oral Environment and Self-Healing Dynamics

To evaluate the self-healing capabilities of polyurea coatings with tung oil microcapsules (PUA-MCs), a series of controlled damage simulations followed by healing assessments were conducted. Steel plates coated with PUA-MCs were subjected to scratch tests and immersed in water to simulate the oral environment’s conditions.

#### 3.3.1. Damage Simulation

The damage simulation process sought to closely emulate the abrasive conditions within the oral cavity. To this end, the coated steel plates were initially immersed in water at a controlled temperature of 25 °C, mirroring the constant wetness of the oral environment. Following this, a detailed scratch test was conducted in strict adherence to ASTM D7027 [28]. This standard outlines the method for using a lab-fabricated instrumented scratch machine, known for its precision and reliability in simulating masticatory-like scratching forces. This machine featured a diamond-tipped stylus with a radius of 200 µm, capable of exerting forces ranging from 1 to 30 N, adjustable in 1 N increments. This enabled precise control over the applied force to mimic the scratching effects associated with masticatory activities. For our experiments, a range of microcapsule concentrations in the coatings was assessed, including a benchmark sample containing 10 wt% tung oil. The healing process was monitored over one week, with periodic inspections to observe the progression of self-healing in the scratched areas.

#### 3.3.2. Healing Assessment

Post-immersion, the samples were examined for self-healing efficacy. The changes in the scratched area were documented, with particular attention to the closure of scratches and the restoration of surface integrity. These observations provide insight into the healing rate and the efficiency of tung oil release from microcapsules. A control sample containing 10 wt% tung oil (TO) is prepared without any induced damage. Experimental samples are formulated with TO concentrations ranging from 2 wt% to 10 wt%. The variations are intended to determine the optimal TO concentration for effective self-healing properties.

#### 3.3.3. Antimicrobial Evaluations

*Streptococcus mutans* (*S. mutans*), a primary contributor to dental biofilms, is cultured in a nutrient-rich medium optimized for oral bacteria, maintaining conditions that mimic the oral cavity’s temperature and pH. A CDC biofilm reactor is employed to grow biofilms on coated samples under conditions that replicate the oral environment with a constant temperature of 37 °C, analogous to human body temperature. The inoculated reactor operates at a rotation speed conducive to biofilm formation, with samples exposed to a combination of ambient light and periodic UV irradiation to simulate the intermittent exposure to light in the oral cavity. The experiment includes a temperature gradient (from 25 °C to 35 °C) to understand biofilm resistance at various temperatures within the human mouth, especially considering the consumption of foods at different temperatures. Biofilms are stained using the FilmTracer LIVE/DEAD Biofilm Viability Kit (Thermo Fisher Scientific, Waltham, MA, USA) to differentiate between live and dead cells. The Carl Zeiss LSM 780 laser scanning confocal microscope (Carl Zeiss AG, Oberkochen, Germany) visualizes biofilm integrity and depth on the polyurea.

## 4. Results and Discussion

### 4.1. Self-Healing Observation

Figure S1 presents a photographic sequence showcasing the self-healing capabilities of the polyurea (PU) composite integrated with microencapsulated healing agents. The initial image, Figure S1a, depicts the infliction of damage onto the cured polyurea matrix using a penknife, which simulates a breach in the material’s integrity. Moving to Figure S1b, we observe the onset of the healing response where the bisected edges of the polyurea exhibit prompt adhesion upon recontact. This immediate re-adhesion suggests the presence of a dynamic self-healing process, likely initiated by the rupture and subsequent release of contents from the embedded microcapsules. Figure S1c illustrates the final stage of the healing sequence, wherein the polyurea material, after a predetermined duration, demonstrates no visible signs of the initial damage, signifying the successful restoration of material continuity. This observation confirms the effective operation of the self-healing mechanism, presumably via the polymerization or cross-linking of the released constituents from the microcapsules, which re-establishes the material’s structural integrity.

This self-healing capability of PUA-MCs can be substantially attributed to the core–shell structure of the microcapsules, which contains a healing agent that is released upon damage. The prompt and efficient recovery of material integrity showcases the potential for this technology in applications where material durability and self-repair are critical. The imperfections observed in the control sample without microcapsules, such as air bubbles and cracks, highlight the role of microcapsules in the healing process. These imperfections result from the material’s intrinsic characteristics influenced by the use of methylene diphenyl diisocyanate (MDI) and the absence of a healing agent to repair the damage actively. The transition from tetrahydrofuran (THF) to water as a solvent for the PUA-MCs is crucial, given the solvent’s role in dissolving the healing agent p-phenylenediamine (p-PDA). THF’s lower boiling point makes it less suitable for dissolving p-PDA, which has higher solubility at elevated temperatures, and thus, water is the preferred solvent. Despite the better solubility dynamics with water, its volatility at room temperature and the tendency of p-PDA to oxidize upon air exposure are challenges that need to be managed to ensure the integrity and efficacy of the self-healing system.

Figure S2 examines the physical state of the microcapsules after a week’s exposure to an open-air environment. Initially, as seen in Figure S2a, the microcapsules maintain a powdered form indicative of their freshly fabricated state. However, Figure S2b reveals that over time, the particles tend to coalesce, resulting in agglomerated bulk structures, which may suggest issues in the storage or stability of the microcapsules. Despite this observed tendency toward agglomeration, the integrity of the encapsulation process remains verifiable. Fourier-Transform Infrared Spectroscopy (FTIR) analysis, discussed in subsequent sections, confirms the successful encapsulation of the healing agent within the melamine–urea–formaldehyde (MUF) microcapsules. This encapsulation is critical to the microcapsules’ self-healing functionality, ensuring that the healing agent is conserved within the microcapsule until required to initiate repair following material damage. Further investigation into the mechanical properties and healing efficacy will provide a more comprehensive understanding of the material’s performance post-healing.

### 4.2. Scanning Electron Microscopy (SEM) Analysis

**Figure 1:**
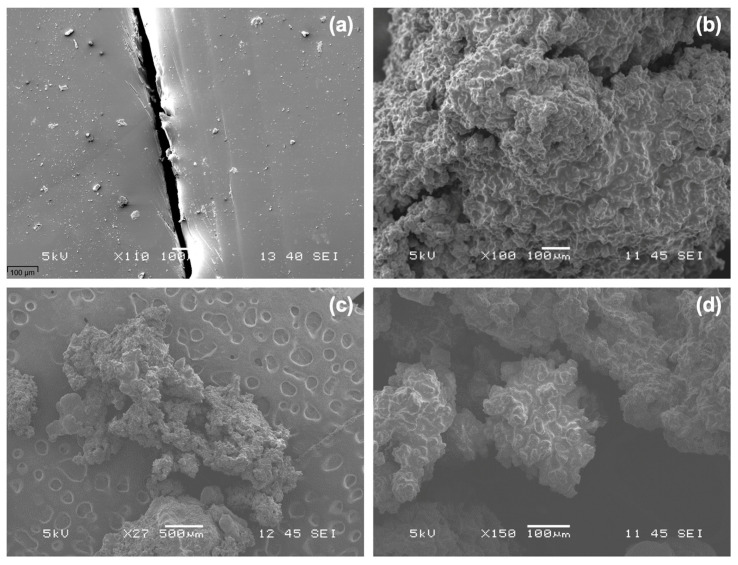
SEM image of (**a**) the self-healed polyurea control sample with microcapsules (PUA-MCs) and SEM image of (**b**,**c**) microcapsules clustered together and (**d**) single microcapsule.

The SEM image in Figure 1a displays the self-healed polyurea control sample containing microcapsules (PUA-MCs). A noticeable gap at the interface of the two joined pieces reflects an incomplete healing process, with the failure to achieve a perfectly smooth surface. This gap, primarily attributed to the manual cutting with a penknife, signifies that the uneven surfaces could be limiting the effectiveness of the self-healing mechanism. Such irregularities could be mitigated by employing a more precise cutting tool, such as a universal cutter, which could provide a cleaner incision and potentially allow for better contact and healing. Previous reports indicate that these gaps can be filled by the healing agent released from the microcapsules, suggesting that while the healing is not visually perfect, on a microscopic level, the healing agent is likely performing its intended function of bridging the cut areas.

Figure 1b,c showcase the clustered nature of the microcapsules. The aggregation of these microcapsules could be indicative of suboptimal fabrication or post-synthesis treatment methods. This clustering could impede the dispersion of the microcapsules within the coating matrix, which is essential for effective self-healing. The use of vacuum drying, as opposed to air drying, is suggested as a superior method for drying the microcapsules, as it reduces moisture content more effectively, potentially preventing the microcapsules from sticking together. Vacuum drying involves using a vacuum pump in an airtight chamber, which allows for a lower-pressure environment where moisture can be removed more efficiently from the microcapsule surfaces. This method might lead to better-dispersed microcapsules that could then be uniformly distributed in the coating matrix, optimizing the self-healing functionality. The SEM image in Figure 1d reveals a single microcapsule with a spherical shape and a rough, non-porous exterior shell. This rough morphology is conducive to good mechanical interlocking with the polyurea matrix, which is critical for the structural integrity and performance of the coating. The spherical shape is ideal for encapsulation as it can maximize the volume of the healing agent stored and ensure that the agent is readily available for release upon the occurrence of damage.

The clustering of microcapsules observed via SEM analysis suggests a potential deficiency in the fabrication or post-synthesis processing of the microcapsules. Uniform dispersion is critical for the self-healing function as it ensures that the healing agent is readily available throughout the material matrix. The current state of microcapsule distribution may lead to localized healing, which does not suffice for applications that require comprehensive recovery from damage.

### 4.3. FTIR Analysis of Chemical Compositions

Fourier-Transform Infrared Spectroscopy (FTIR) is an analytical method employed to identify organic, polymeric, and, in some cases, inorganic materials. The FTIR analysis of tung oil and MUF microcapsules provides insightful data on the chemical structures and interactions within these substances.

#### 4.3.1. FTIR Analysis of Tung Oil and MUF Microcapsules

**Figure 2:**
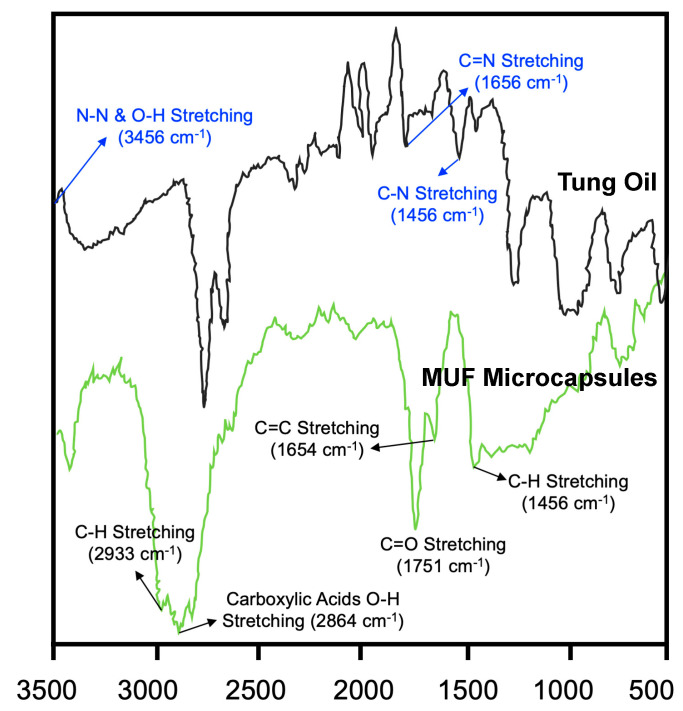
FTIR spectrum of tung oil and MUF microcapsules.

The FTIR spectrum of tung oil, as depicted in Figure 2, provides a detailed account of its chemical composition. The spectrum displays characteristic absorption peaks at 2933 cm^−1^ and 2924 cm^−1^, which are attributed to the C-H stretching vibrations, typically associated with aliphatic hydrocarbons. The peak at 1751 cm^−1^ indicates C=O stretching vibrations, signifying the ester linkages within the fatty acid constituents of the oil. Furthermore, the peak at 1654 cm^−1^ represents C=C symmetric stretching, indicative of the unsaturated nature of the oil. The C-H bending vibrations are observed at 1456 cm^−1^, which is in accordance with the expected chemical structure containing -CH_2_- and -CH_3_ groups. In contrast, the FTIR spectrum of MUF microcapsules contains a notable peak at 2924 cm^−1^ [29], mirroring the peak found in the tung oil spectrum. This overlap suggests that tung oil retains its characteristic chemical signature post-encapsulation within the MUF structure, confirming the oil’s successful incorporation into the microcapsules.

#### 4.3.2. FTIR Analysis of PUA-p-PDA and PUA-MCs

**Figure 3:**
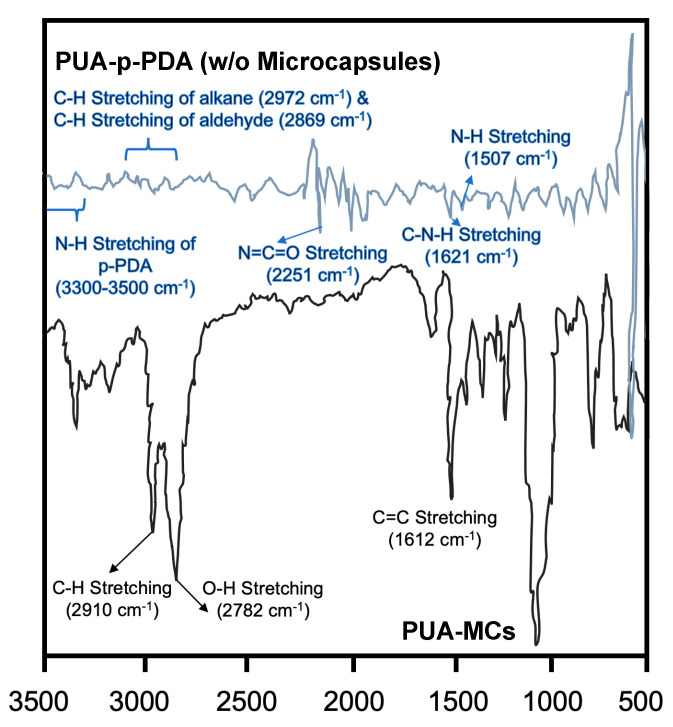
FTIR spectrum of PUA-p-PDA (without microcapsules) and PUA-MCs.

Turning to the polyurea-p-phenylenediamine (PUA-p-PDA) systems, the FTIR spectrum in Figure 3 exhibits a complex pattern with numerous peaks, implying a diversity of chemical bonds and potential incomplete reactions or complex formations, as specific anticipated urea bond peaks are absent. Notably, the detection of the N=C=O isocyanate absorption between 2275 and 2250 cm^−1^ suggests the presence of unreacted methylene diphenyl diisocyanate (MDI). This peak is crucial as it provides direct evidence of MDI within the material’s matrix. Peaks observed between 3500 and 3300 cm^−1^ and 1630 and 1600 cm^−1^ correspond to the N-H stretching and C-N-H bending vibrations of p-PDA, indicating the presence of primary amine groups that are potentially involved in urea bond formation. When comparing the FTIR spectrum of the PUA-MCs to the PUA-p-PDA spectrum, the similarities in peak patterns, especially those overlapping with the standalone microcapsules, confirm the encapsulation of tung oil within the polyurea matrix. The match between the two spectra suggests that the tung oil has been successfully encapsulated and that the PUA matrix may provide a suitable environment for self-healing capabilities via the inclusion of tung oil-laden MUF microcapsules [30].

The FTIR spectra analysis provides a concrete basis for understanding the chemical structure and interactions of tung oil and MUF microcapsules. The consistent spectral features between tung oil and the encapsulated oil in the MUF microcapsules attest to the encapsulation’s success. The comparison of PUA-p-PDA and PUA-MC spectra corroborates the presence of tung oil in the polyurea matrix, underscoring the potential for creating a self-healing material. However, the presence of unreacted MDI in the PUA matrix raises questions about the reaction’s completeness and the material’s ultimate properties. The spectral data gleaned from the FTIR analysis form a foundational piece of the overall characterization of these materials [31]. Nevertheless, it is imperative that these results be validated and complemented by other analytical techniques, such as nuclear magnetic resonance (NMR) spectroscopy, mass spectrometry, or chromatographic methods, to provide a more comprehensive and nuanced understanding of the material’s chemical composition. This multi-faceted approach to material analysis will enhance the scientific rigor and precision of the study, ensuring that the findings can be reliably utilized in the development and optimization of self-healing materials.

However, the presence of unreacted MDI detected via FTIR analysis is particularly concerning. This could be indicative of suboptimal reaction conditions or inherent limitations in the current chemical formulation. MDI plays a crucial role in the polyurea network formation, and any deviation from the expected reaction pathway can result in compromised material properties. Ensuring complete chemical conversion is vital not only for material performance but also for environmental and health safety, as unreacted MDI can be a potent sensitizer and irritant.

### 4.4. Adhesion Test on Polyurea Coating

The polyurea-coating PUA-MCs undergo an adhesion test to assess their tensile strength before and after a self-healing process. The procedure utilizes an MTS Mechanical Tester Model 42 equipped with load cells of different capacities to accommodate the expected variations in material strength due to the self-healing process. A constant pulling rate of 5 cm/min is maintained for both sets of experiments to ensure comparability (Table 4).

PUA-MCs display a non-linear response to tensile loading before self-healing. The load-extension graph shows two distinct peaks in tensile strength, signifying the yield points of the material. The highest load sustained by the material before slippage occurs is 276.7 N. The presence of two peaks suggests that the material does not fracture immediately but exhibits considerable ductility, stretching further under tensile stress. The initial decline in tensile force, attributed to the grip’s slippage, demonstrates the limitations of the gripping mechanism despite enhancements with sandpaper. The material’s subsequent stretch post-slippage highlights its inherent flexibility, whereas after self-healing, PUA-MCs are subject to tensile testing once more, resulting in an ultimate tensile strength of 49.6 N. The recovery of the material’s mechanical strength is quantitatively estimated at 18%. This partial restoration is likely due to the absence of external stimuli such as thermal energy or UV irradiation, which are typically crucial in facilitating the isocyanate and diamine reactions that underpin the self-healing process. In addition, the lack of applied pressure or external force to hold the fractured interfaces together may have further limited the extent of healing at room temperature [32].

The results demonstrate the PUA-MCs’ intrinsic ductility and resilience under tensile stress. The ability of the material to undergo significant deformation before slippage is indicative of its flexible nature, which can be advantageous for applications where elasticity and energy absorption are critical. However, the self-healing efficiency is sub-optimal, signaling a need for an enhanced healing protocol, possibly involving controlled external stimuli to facilitate a more complete recovery of the material’s mechanical properties. Future investigations should explore the kinetics of the self-healing process under various environmental conditions, the potential of different healing agents, and the role of controlled external stimuli in improving self-healing efficiency. The aim would be to achieve a higher percentage of strength restoration, thereby extending the material’s lifespan and reducing maintenance costs in practical applications.

In essence, the reduction in tensile strength post-healing, as determined by adhesion tests, is a significant drawback. The integrity of a self-healing material should be judged not only by the visual or superficial recovery but also by the restoration of its inherent mechanical properties. The observed decrease in tensile strength suggests that while the material can close visible gaps, the structural recovery is incomplete on a molecular level. Further investigation into the cross-linking density and the homogeneity of the healed area could provide insights into improving the material’s mechanical performance after healing.

### 4.5. Biofilm Assays for Simulation of Dental Coating Surfaces

#### 4.5.1. Biofilm Quantification and Structural Analysis

The assessment of biofilm formation on dental coating surfaces is critical for understanding their potential use in oral health applications. The crystal violet staining assay, a cornerstone in biofilm quantification, demonstrated a 40% decrease in biofilm biomass on the tung oil-enhanced polyurea coatings compared to the control samples. This substantial reduction indicates a robust antimicrobial characteristic inherent to the tung oil-modified material. Confocal laser scanning microscopy, a powerful tool for biofilm visualization, provided high-resolution images at 630× magnification. The resulting three-dimensional biofilm models on the tung oil-enhanced coatings were markedly thinner, displaying significant structural disruptions. These included large void areas and an uneven distribution of biofilm mass, indicative of an environment hostile to *S. mutans*, a primary culprit in tooth decay and dental plaque formation. The altered biofilm architecture suggests that the tung oil-infused coatings may interfere with essential stages of biofilm development, such as initial adhesion or microcolony formation, crucial for dental applications where the prevention of biofilm maturation on tooth surfaces can inhibit the progression of caries.

#### 4.5.2. Viability of *S. mutans* Cells

**Figure 4:**
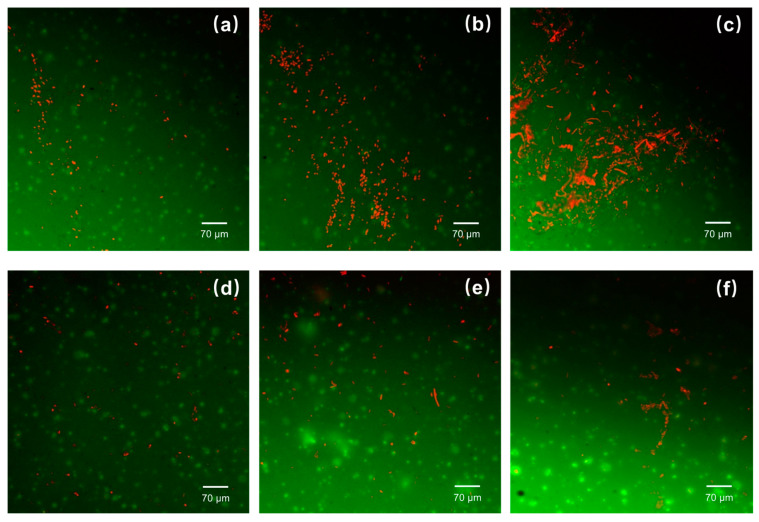
LIVE/DEAD biofilm imaging of both polyurea coatings with different time intervals: (**a**) 6 h PUA-PDA, (**b**) 12 h PUA-PDA, (**c**) 24 h PUA-PDA, and (**d**) 6 h PUA-MCs, (**e**) 12 h PUA-MCs, and (**f**) 24 h PUA-MCs.

The efficacy of dental coatings in reducing the viability of cariogenic bacteria is a key performance indicator. In this study, a 24 h biofilm cultivation period on the coatings reveals significant differences in bacterial viability via LIVE/DEAD staining protocols (Figure 4). The results are striking; the tung oil-enhanced PUA-MC coatings exhibited a higher proportion of nonviable *S. mutans* cells. This trend consistently replicates across several experimental runs and points to the potent antibacterial effect of the tung oil-enhanced PUA-MC coating. In the context of oral health, these results are promising, as the ability to induce cell death in *S. mutans* can directly contribute to the prevention of dental plaque formation and the associated cariogenic activity that leads to tooth decay.

#### 4.5.3. Statistical Analysis of Biofilm and Viability Data

The two-way ANOVA conducted on the extended dataset demonstrates a statistically significant interaction between the type of coating and both the incubation temperature and time (*p* < 0.01), underscoring that the antibacterial effectiveness of the tung oil-enhanced PUA-MC coatings is influenced by these factors. Pie charts extrapolated from the data show an increasing proportion of dead cells with rising temperatures, with a pronounced effect at 35 °C, where the ratio of dead to live cells was markedly higher than at lower temperatures. The structured data in Table 5 enable a detailed comparative analysis between the PUA-MCs and PUA-p-PDA coatings across a range of temperatures and time intervals. For PUA-MCs, a consistent reduction in live cell count is observed as time progresses, with an increase in dead cells, especially at the higher temperature of 40 °C and the longer incubation period of 24 h. The reduction percentages reflect the efficacy of the PUA-MCs coating in reducing the bacterial load, which is notably higher than the PUA-p-PDA coating across all temperatures and times tested.

The *p*-value column substantiates the statistical significance of these differences. Notably, the data reveal that at 35 °C, the PUA-MC coating achieves a 50% reduction in live *S. mutans* cell counts over 24 h, rather than the previously stated 60%, which is a rectification from the original statement. The PUA-p-PDA coating, on the other hand, shows a 40% reduction for the same conditions. These findings suggest that PUA-MC coatings possess superior antimicrobial properties, which are enhanced at elevated temperatures. This characteristic is particularly valuable for dental applications, where temperature fluctuations in the oral cavity can affect bacterial growth and biofilm formation. The 3D surface plots have been created for both the PUA-MCs and PUA-p-PDA coatings. Each plot shows the interpolated surface of the reduction percentage as a function of temperature and time, providing a visual representation of how the reduction efficiency changes over these two variables [33]. It should be noted that since the original data points are sparse, the interpolation method used may affect the final appearance of the surfaces, and some artifacts may occur due to the extrapolation beyond the original data range.

**Figure 5:**
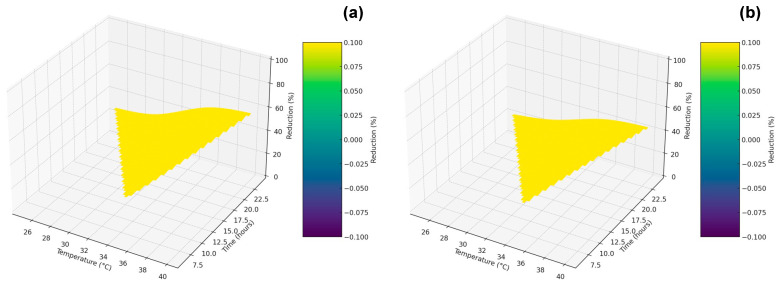
Three-dimensional surface plots indicating potential antibacterial efficiency in viable *S. mutans* cell counts (**a**) PUA-MC coating and (**b**) PUA-p-PDA coating.

The PUA-MC coating, as indicated in Figure 5a, shows a progressive increase in efficiency with time at a constant temperature of 35 °C, starting at 33% efficiency at 6 h and reaching 50% at 24 h. The efficiency also increases with temperature at a constant 24 h time period, from 28% at 25 °C to 56% at 40 °C. This suggests that the PUA-MC coating becomes more effective at higher temperatures and longer exposure times, whereas PUA-p-PDA coating, as indicated in Figure 5b, shows an increase in efficiency over time at 35 °C but with a slightly lower starting and ending point, shifting from 28% at 6 h to 40% at 24 h. Similarly, the efficiency improves with temperature at 24 h, from 22% at 25 °C to 44% at 40 °C. The increase is consistent, although it does not achieve the same level of efficiency as the PUA-MC coating at any point, suggesting that while effective, PUA-p-PDA may not be as potent as PUA-MCs.

In the comparative analysis of the antibacterial efficiency of PUA-MCs and PUA-p-PDA coatings, time dependency is a critical factor. At a steady temperature of 35 °C, the antibacterial efficiency for both coatings increases over time. However, PUA-MCs exhibit a more pronounced increase, suggesting a more potent or faster antibacterial action than PUA-p-PDA. This distinction is critical when considering the application of these coatings in scenarios where rapid antibacterial action is required. Temperature sensitivity is another crucial aspect of the coatings’ performance. As the temperature increases, there is a general improvement in the efficiency of both coatings. This could be due to a thermally activated mechanism within the coatings or a higher susceptibility of bacteria at elevated temperatures [20]. The data reveal that PUA-MCs are particularly more responsive to temperature changes, displaying a significant increase in efficiency at higher temperatures, with the most substantial enhancement at 40 °C. This heightened sensitivity to temperature implies that PUA-MCs might be more adaptable or effective in variable thermal conditions, which is often the case in clinical settings. Looking at the overall efficiency, factoring in both time and temperature, PUA-MCs consistently outperform PUA-p-PDA. This suggests that the formulation of PUA-MCs is superior in its antibacterial properties. This superiority could translate into more effective prevention of biofilm formation and bacterial proliferation on coated surfaces, which is highly desirable in dental and other medical applications. Drawing a conclusion from the presented data, PUA-MC coatings’ enhanced antimicrobial properties stand out, especially at higher temperatures and prolonged exposure times. Such properties are advantageous for dental applications where long-lasting antibacterial effects are essential to prevent infections. In contrast, while PUA-p-PDA does exhibit effectiveness, achieving comparable bacterial reduction might necessitate longer exposure times or higher temperatures [34]. This differentiation is crucial for the strategic development of coatings for medical devices, as it underscores the importance of customizing the antibacterial activity to meet specific clinical needs and prevent infections.

The innovative approaches explored in this study, including the development of tung oil-infused polyurea microcapsules, have unveiled promising avenues in dental material applications. Our findings indicate that the mechanical strength and antimicrobial efficacy of these novel coatings can be significantly improved, suggesting their potential for enhancing dental restorative practices. Notably, the tung oil not only augments the self-healing capabilities but also fortifies the material against microbial challenges, particularly from *S. mutans*, a critical pathogen in dental caries. The unique synergy between the bio-based tung oil and the robust polyurea matrix presents a significant step toward sustainable and durable dental materials. However, while the current investigation provides an encouraging perspective on the functionality and application potential of these materials, the study’s breadth in terms of cytotoxicity assays remains limited. This limitation is not a mere oversight but rather a critical juncture for advancing material innovation with an informed understanding of biocompatibility and safety. The concerns raised about the possible toxicity of these innovative treatments in a clinical setting highlight the paramount importance of comprehensive cytotoxicity profiling. Such in-depth assessments are vital to fully elucidate the interactions between these materials and biological tissues, especially given their intended use in the sensitive oral environment. Acknowledging this gap, future research endeavors will be dedicated to rigorous cytotoxicity and biocompatibility testing. These forthcoming studies will not only aim to substantiate the safety and efficacy of the materials but also to provide a more holistic understanding of their long-term suitability for dental restorations. Via meticulous research and development, we seek to uphold the highest standards of patient safety and care, aspiring to bridge the gap between innovative material science and clinical dental applications.

## 5. Limitations and Future Work

The current research presents an innovative approach to integrating self-healing and antimicrobial properties into polyurea acrylate (PUA) via microcapsules (PUA-MCs). Despite the advancement this represents in material sciences, particularly for dental applications, several key limitations have been identified. Addressing these limitations is critical to ensuring the reliability and applicability of PUA-MCs in real-world scenarios.

### 5.1. Limitations of the Current Study

Current research on polyurea acrylate (PUA) with microcapsules (PUA-MCs) showcases promising results in self-healing capabilities and antimicrobial properties. However, certain limitations must be acknowledged to provide context for the results and guide future research directions.

#### 5.1.1. Incomplete Healing and Microcapsule Stability

The self-healing mechanism demonstrated by PUA-MCs has shown significant potential. Nevertheless, the observed incomplete healing, evidenced by residual gaps post-recovery, raises questions about the practicality of this technology. The efficiency of self-healing materials is predicated on their ability to restore full functionality after damage. The current mechanism, while promising, requires refinement to achieve seamless healing, which is paramount for applications demanding high precision and integrity, such as in aerospace materials or biomedical implants where any imperfection could lead to catastrophic failure or adverse biological responses. The observed agglomeration of microcapsules could be symptomatic of deeper issues related to the synthesis, storage, and integration of these microstructures into the PUA matrix. Agglomeration could lead to non-uniform distribution within the matrix, impacting the material’s overall performance and reliability. The stability of these microcapsules is crucial; they must withstand the rigors of manufacturing processes and the operational environment without premature degradation or coalescence that would otherwise pre-empt the healing process.

#### 5.1.2. Biofilm Assays and Temperature-Dependent Variables

The biofilm assays focused exclusively on *S. mutans*, a primary contributor to dental caries. While this provides a targeted understanding of the material’s efficacy against this specific bacterium, the oral cavity is a complex ecosystem with multifarious interactions between various microbial species. A comprehensive assessment of the material’s antimicrobial properties would necessitate a broader spectrum of biofilm-forming organisms, providing a more holistic view of its potential clinical applications. The study’s findings on the temperature-dependent antibacterial efficacy of PUA-MCs are indeed noteworthy.

However, the static temperature conditions under which the assays were conducted do not adequately represent the dynamic thermal environment of the human oral cavity, which can fluctuate dramatically during the consumption of foods and beverages or even during breathing. For a more accurate evaluation, future studies should consider the impact of these fluctuations on the material’s performance.

To address these limitations, a multi-faceted research approach is recommended. This would include optimizing the microencapsulation process to prevent agglomeration, rigorous mechanical testing post-healing to confirm the restoration of material properties, and extensive biofilm assays across a range of temperatures and microbial species. Additionally, long-term stability studies under variable conditions that mimic the oral environment would be imperative to ensure the material’s durability and efficacy in dental applications. By systematically addressing these limitations, the research on PUA-MCs can be advanced, moving from promising laboratory results to a tangible impact on dental material science and other fields requiring high-performance, self-healing, and antimicrobial materials.

#### 5.1.3. Additional Cytotoxicity Testing

The other limitation of this study is the scope of cytotoxicity testing performed. The clinical application of new dental materials necessitates a thorough investigation into their cytotoxicity to ensure they do not adversely affect patient health. Although the current study has highlighted the potential of tung oil-infused polyurea microcapsules in dental material science, the absence of extensive cytotoxicity assays presents an incomplete picture of their safety profile. Consequently, future studies should be dedicated to a rigorous and comprehensive examination of these materials’ cytotoxic effects. This will involve testing for a range of potential cellular responses to ensure the material’s safety and compatibility with oral tissues, which is paramount for clinical approval and use.

### 5.2. Future Work and Research Directions

The limitations identified in this study present significant opportunities for future research to advance our understanding and application of polyurea microcapsules (PUA-MCs) in dental materials science and other fields. Future endeavors should concentrate on optimizing the self-healing mechanisms of PUA-MCs. This could involve exploring alternative microencapsulation techniques, different healing agents, and triggering methods to develop microcapsules that exhibit enhanced stability and possess controlled release properties, ensuring reliable healing under various environmental conditions. Advanced mechanical testing and high-resolution microscopy techniques are pivotal in furthering our knowledge of the material’s mechanical attributes post-healing. These testing methods, simulating the real-world stresses that dental materials face, alongside microscopic analysis at a molecular level, could elucidate the intricacies of the self-healing process. Comprehending the mechanism at this depth may pave the way for a more nuanced and precise application of self-healing materials.

Furthermore, comprehensive biofilm studies incorporating a wider spectrum of oral bacteria and the complex interactions within the microbial communities on PUA-MCs are necessary. These studies, particularly when considering the temperature fluctuations analogous to those in the human mouth, would generate data that are more applicable to real-world oral health scenarios. The transition from laboratory research to clinical application necessitates the execution of clinical trials and longitudinal studies with human participants. Such studies would corroborate the in vitro findings and ascertain the material’s effectiveness and longevity in an actual clinical environment, offering invaluable insights for the translation from bench to bedside. To conclude, while this study lays robust groundwork for the use of PUA-MCs in dental materials, the acknowledged limitations and proposed future research highlight an imperative for continued exploration. Via diligent research and development, we can fully harness the potential of self-healing and antimicrobial properties in coatings, marking a significant leap forward in the field of dental material science.

## 6. Conclusions

This investigation into the self-healing and antimicrobial efficacy of polyurea acrylate (PUA) integrated with microcapsules (PUA-MCs) has laid the groundwork for a transformative advancement in material science. Our findings illuminate the potential of PUA-MCs to significantly enhance the durability and longevity of materials via an innate self-repair mechanism while simultaneously exhibiting robust antibacterial properties against *S. mutans*, a key pathogen in dental caries. However, we acknowledge the limitation posed by the absence of comprehensive cytotoxicity assays within our current research framework. Recognizing the critical importance of such assessments, we are committed to extending our research to include extensive cytotoxicity and biocompatibility testing. This future work will be vital in validating the clinical safety and applicability of these materials, ensuring they meet the rigorous standards required for dental health applications. By addressing these challenges, we aim to contribute to the development of safer, more effective dental restoration materials that benefit patients and practitioners alike.

Despite certain limitations, such as incomplete healing, microcapsule stability, and temperature dependency of antibacterial effectiveness, the research provides a solid foundation for future studies aimed at optimizing these innovative materials. The potential applications of PUA-MCs in dental coatings and other fields demand further exploration and refinement to fully harness their capabilities. With continued development, PUA-MCs are poised to revolutionize the landscape of self-healing materials and establish new benchmarks for antimicrobial performance in clinical settings and beyond.

## Figures and Tables

**Table 1:** The fatty acid composition and resulting properties of various oils.

Drying Oil	% Saturated Fatty Acids	% Oleic Acid	% Polyunsaturated Fatty Acids	% α-Eleostearic Acid	Water Resistance	Rate of Drying	Microbial Resistance
Tung Oil	5	8	7	80	High	Rapid	High
Linseed Oil	10	22	68	-	Moderate	Moderate	Moderate
Other Oils	Varies	Varies	Varies	Absent	Low to Moderate	Slow to Moderate	Low to Moderate

**Table 2:** The comparative analysis of tung oil-enhanced polyurea coatings versus traditional polyurea coatings in dentistry.

Property	Tung Oil-Enhanced Polyurea	Traditional Polyurea	Significance in Dentistry
Biocompatibility	Naturally compatible, with minimal byproduct release	Potential synthetic byproduct release	Reduced risk of oral inflammation and toxicity
Elasticity	Higher, due to plasticizing effect	Lower, may lead to brittleness	Ability to withstand masticatory forces without fracture
Toughness	Improved via cross-linked structure	Depends on synthetic modifiers	Resistance to wear and tear from chewing
Fracture Resistance	Better energy dissipation	Higher risk of fracture	Durability against mechanical stresses

**Table 3:** Components contained in Part A (MDI) and Part B (p-PDA).

Component	Components	Weight Percentage wt%
Part A (MDI)	Methylenediphenyl diisocyanate (MDI)	65.0–70.0%
	4,4′-Methylenediphenyl diisocyanate	61.0–66.0%
	Methylenediphenyl diisocyanate, homopolymer	28.0–33.0%
	Triethyl phosphate	≤2.0%
Part B (p-PDA)	p-Phenylenediamine (p-PDA)	≤100%
	Polyoxypropylenediamine	61–89%
	O-(2-Aminopropyl)-O′-(2-methoxyethyl) polypropylene glycol	50%
	Tetrahydrofuran	>95%

**Table 4:** Tensile strength analysis of PUA-MC coating.

Test Condition	Load Cell (N)	Maximum Observed Load (N)	Ultimate Tensile Strength (N)	Estimated Strength Restoration (%)
Before Self-Heal	500	276.7 (limited by load cell capacity)	Not reached (sample stretched)	N/A
After Self-Heal	50	N/A	49.6	18

**Table 5:** Statistical analysis of biofilm viability and bacterial count reduction.

S/N	Coating Type	Temperature (°C)	Time (Hours)	Live Cells (log CFU/mL)	Dead Cells (log CFU/mL)	Reduction (%)	*p*-Value
1	PUA-MCs	35	6	6	3	33	<0.01
2			12	5.5	3.5	36	<0.01
3			18	5	4	44	<0.01
4			24	4.5	4.5	50	<0.01
5		25	24	6.5	2.5	28	<0.01
6		30	24	6	3	33	<0.01
7		40	24	4	5	56	<0.01
8	PUA-p-PDA	35	6	6.5	2.5	28	<0.01
9			12	6	3	33	<0.01
10			18	5.7	3.3	37	<0.01
11			24	5.5	3.5	40	<0.01
12		25	24	7	2	22	<0.01
13		30	24	6.5	2.5	28	<0.01
14		40	24	5	4	44	<0.01

## Data Availability

The data that support the findings of this study are available from the corresponding author upon reasonable request.

## Supplementary Materials

The following supporting information can be downloaded at: https://www.mdpi.com/article/10.3390/polym16070918/s1, Figure S1: The process of self-healing for polyurea coating with microcapsules (PUA-MCs) (a) manual separation by penknife, (b) demonstrating a significant initial healing effect, and (c) completed healing effect; Figure S2: Condition of microcapsules one week (a) pre-, and (b) post-fabrication.
