# Supplementary material for: Enhancing Dental Material Performance: Tung Oil-Infused Polyurea Microcapsule Coatings for Self-Healing and Antimicrobial Applications
Source: Polymers (Basel). 2024 Mar 27;16(7):918. doi: 10.3390/polym16070918 (PMC11013920; doi:10.3390/polym16070918)
Supplement: Supplementary file 1 [file polymers-16-00918-s001.zip › polymers-2907690-supplementary.pdf]

Supplementary Materials

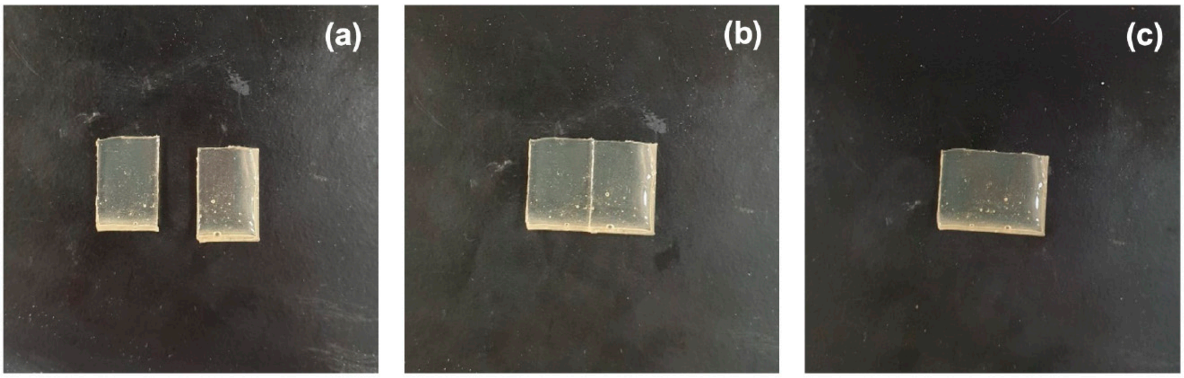

Figure S1. The process of self-healing for polyurea coating with microcapsules (PUA-MCs) (a) manual separation by penknife, (b) demonstrating a significant initial healing effect, and (c) completed healing effect.

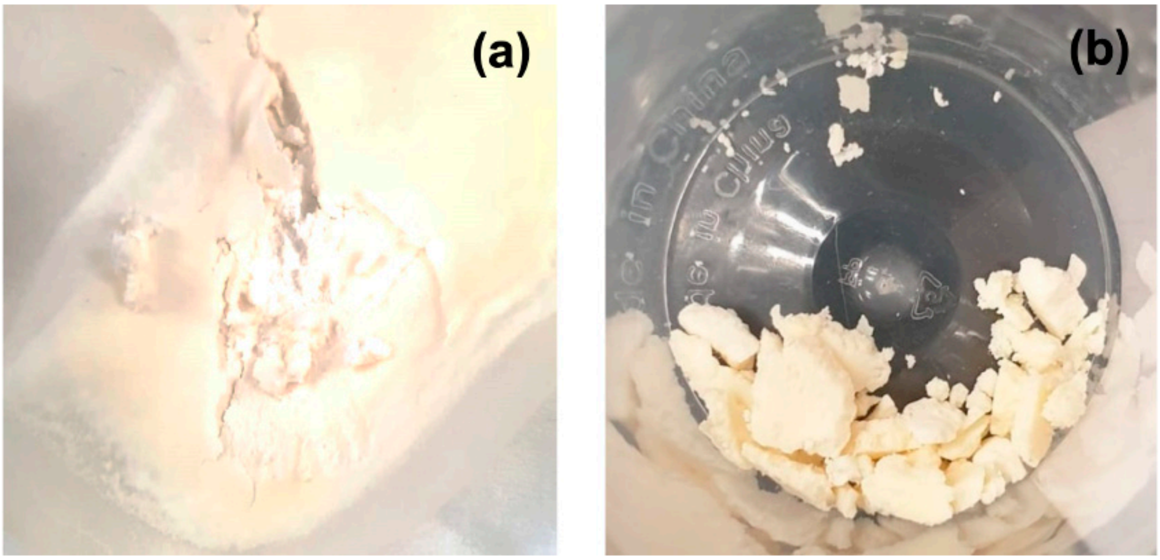

Figure S2. Condition of microcapsules one week (a) pre-, and (b) post-fabrication.
